# Longitudinal Profiling of DNA Methylation Reveals Age‐Varying CpG Sites and Novel Insights Into Aging Heterogeneity

**DOI:** 10.1111/acel.70362

**Published:** 2026-01-02

**Authors:** Xueqing Jia, Hongwei Chen, Liming Zhang, Jingyun Zhang, Yuyang Zheng, Weicheng Wu, Xuehui Sun, Xiaoyan Jiang, Yin Yao, Li Jin, Xiaofeng Wang, Zuyun Liu

**Affiliations:** ^1^ Center for Clinical Big Data and Analytics Second Affiliated Hospital, Department of Big Data in Health Science School of Public Health, Zhejiang Key Laboratory of Intelligent Preventive Medicine Zhejiang University School of Medicine Hangzhou Zhejiang China; ^2^ Department of Health Policy and Management Yale School of Public Health New Haven Connecticut USA; ^3^ Department of Epidemiology and Health Statistics, School of Public Health Hangzhou Medical College Hangzhou Zhejiang China; ^4^ Human Phenome Institute, Zhangjiang Fudan International Innovation Centre, Fudan University Shanghai China; ^5^ Fudan University‐The People's Hospital of Rugao Joint Research Institute of Longevity and Ageing Rugao Jiangsu China; ^6^ Key Laboratory of Arrhythmias, Ministry of Education, Department of Pathology and Pathophysiology, School of Medicine Tongji University Shanghai China; ^7^ Department of Biostatistics and Computational Biology, School of Life Sciences Fudan University Shanghai China; ^8^ State Key Laboratory of Genetic Engineering, Human Phenome Institute, Center for Evolutionary Biology, and School of Life Sciences Fudan University Shanghai China; ^9^ Human Phenome Institute, Fudan University Shanghai China; ^10^ National Clinical Research Center for Aging and Medicine, Huashan Hospital, Fudan University Shanghai China; ^11^ State Key Laboratory of Genetic Engineering, Collaborative Innovation Center for Genetics and Development, School of Life Sciences Fudan University Shanghai China

**Keywords:** aging heterogeneity, DNA methylation variability, epigenetic clock, longitudinal study, organ systems aging

## Abstract

Age‐varying DNA methylation sites reflect increasing interindividual epigenetic divergence during aging, offering insights into health heterogeneity and potential for personalized interventions. Leveraging longitudinal DNA methylation data (3 waves over 5 years) from 135 relatively healthy Chinese older adults in the Rugao Longitudinal Ageing Study, we systematically characterized dynamic DNA methylation changes with age via mixed‐effects modeling, identifying 125,353 age‐associated (i.e., sites showing significant shifts in average methylation levels with age) and 3145 age‐varying CpG sites (i.e., sites showing significant interindividual variability in methylation trajectories with age). Functional analysis revealed distinct enrichment profiles: age‐associated CpG sites were enriched in nervous system development, cell signaling, and disease‐related pathways, whereas age‐varying CpG sites were enriched in cell adhesion, synaptic organization, and organ morphogenesis pathways. Notably, both categories showed significant enrichment in nervous system‐related pathways, such as regulation of nervous system development and neuronal cell body. Established epigenetic clocks (e.g., HannumAge) were significantly enriched for age‐associated CpG sites but not for age‐varying sites. Furthermore, we quantified the pace of aging across eight major organ systems and identified 925 significant associations between organ‐specific pace of aging and longitudinal methylation change rates at age‐varying CpG sites. Pathway enrichment analysis revealed organ system‐relevant biological functions—CpG sites associated with a given organ system were often enriched in pathways relevant to that system's function—with additional evidence of cross‐system enrichment. Together, our findings elucidate the role of methylation variability in multi‐organ systems aging and its potential for revealing mechanisms of aging heterogeneity and guiding precision monitoring and interventions.

## Graphical abstract

We longitudinally profiled DNA methylation in 135 relatively healthy older adults from the RLAS study, identifying 3145 CpG sites exhibiting increasing inter‐individual variability with age and 125,353 sites exhibiting significant changes in average methylation levels with age. These two CpG categories showed distinct functional enrichments with overlapping relevance to nervous system pathways. Furthermore, we identified significant associations between longitudinal change rates at these sites and the pace of aging across eight major organ systems, highlighting the importance of methylation variability in shaping aging heterogeneity.

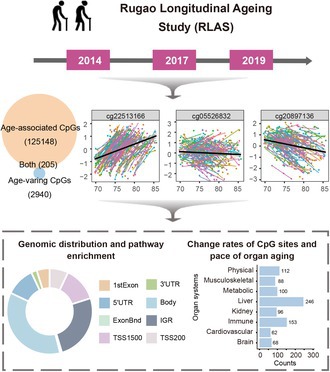

## 1 Introduction

DNA methylation is a key epigenetic mechanism that regulates diverse genome functions such as gene expression and chromatin stability, which may intervene in physiological events (Ji et al. 2010). Methylation patterns change dynamically during aging, influenced by genetic, environmental, and stochastic factors (Jaenisch and Bird 2003). These changes are broadly categorized as age‐associated CpG sites, reflecting shifts in average methylation levels, and age‐varying CpG sites, reflecting increases in interindividual variability (Slieker et al. 2016; Wang, Pedersen, and Hägg 2018). Understanding both types of changes is essential for elucidating the epigenetic mechanisms underlying biological aging and their potential implications for health and diseases.

Previous studies have primarily focused on age‐associated DNA methylation changes and their associations with the onset and progression of age‐related diseases (e.g., cancer and other chronic conditions), while also using them to develop epigenetic clocks, highlighting their potential as biomarkers and therapeutic targets for aging and health (Alisch et al. 2012; Bell et al. 2012; Hannum et al. 2013; Rutledge et al. 2022). In contrast, research on age‐varying DNA methylation patterns remains scarce. Such sites capture divergence in methylomes across individuals over time, which may underlie heterogeneity in health‐related phenotypes (Deelen et al. 2013; Wang, Pedersen, and Hägg 2018). Most existing evidence, however, comes from cross‐sectional studies with small sample sizes and limited genome‐wide coverage, which cannot adequately capture age‐related changes in methylation variability across individuals—a dynamic that can be best assessed using longitudinal data (Fernández et al. 2015; Talens et al. 2012; Slieker et al. 2016; Sluiskes et al. 2024). Moreover, the physiological relevance of these age‐varying methylation changes—particularly their ability to capture differences in the pace of aging across multiple organ systems—remains poorly characterized.

Here, leveraging longitudinal DNA methylation data (3 waves over 5 years) from 135 relatively healthy Chinese older adults in the Rugao Longitudinal Ageing Study (RLAS), we systematically characterized dynamic DNA methylation changes with aging, identifying 3145 age‐varying CpG sites and exploring their underlying functional implications. Furthermore, we examined the associations between longitudinal change rates at these sites and the pace of aging across eight major organ systems, highlighting the importance of methylation variability in shaping aging heterogeneity and its potential to guide personalized interventions.

## 2 Methods

### 2.1 Study Populations

**FIGURE 1:**
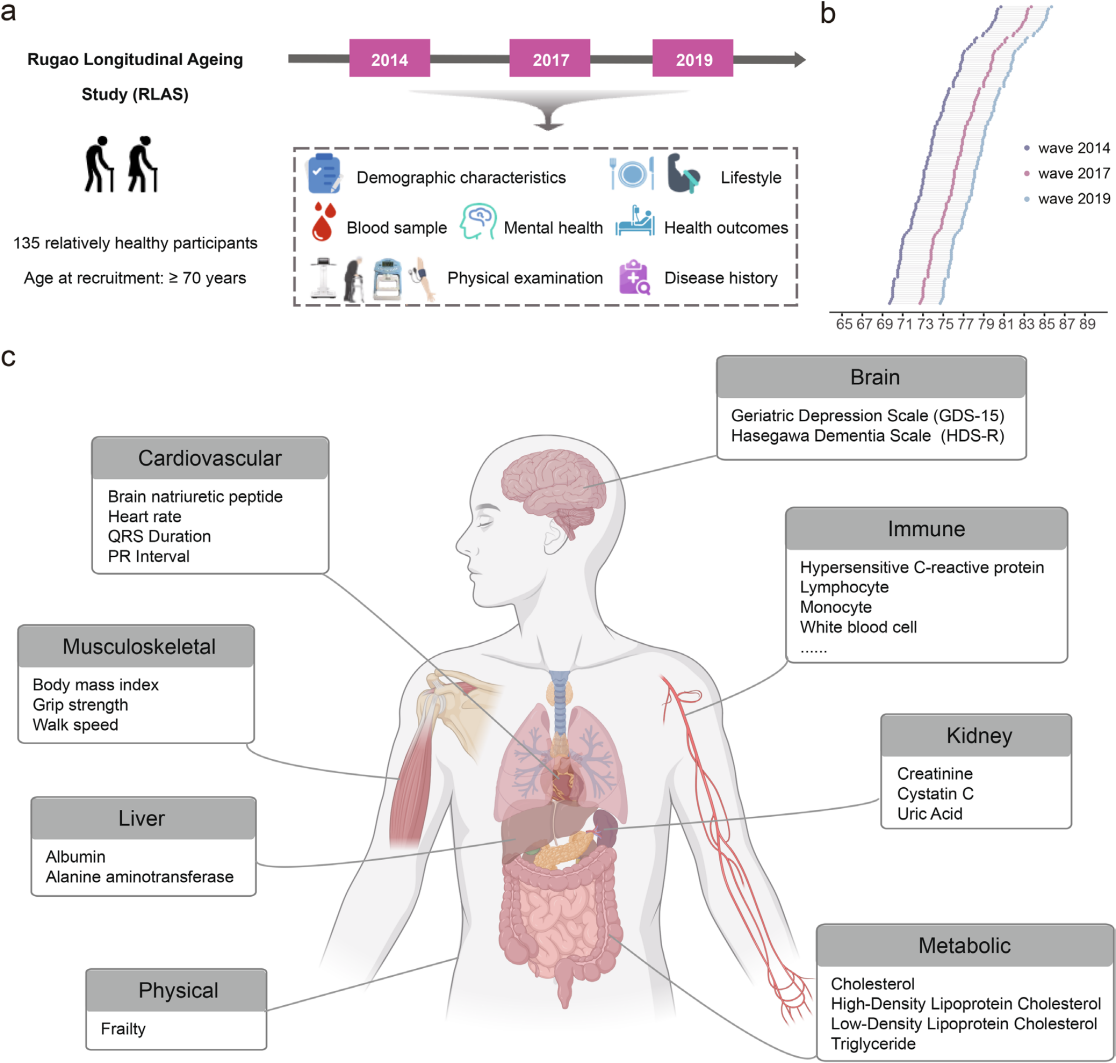
Overview of study design. (a, b) This study included 135 relatively healthy older adults aged ≥ 70 years who participated in all three survey waves (waves 2014, 2017, and 2019) of the Rugao Longitudinal Ageing Study (RLAS). Comprehensive longitudinal profiling of both macro‐ and micro‐phenotypes was conducted. (c) Organ systems for which the pace of aging was established using organ systems‐specific phenotypes. Key phenotypes are listed below each organ system. Original elements of Figure 1c were downloaded from BioRender.com and subsequently modified for clarity.

The RLAS is a population‐based longitudinal cohort of older adults in Rugao, Jiangsu Province, China. A detailed description of the RLAS has been published previously (Liu et al. 2016). The RLAS initially recruited 1788 participants aged ≥ 70 years during the baseline survey in November 2014. Follow‐up assessments were conducted in May 2017 (3‐year follow‐up) and subsequently in November 2019 (5‐year follow‐up). Trained medical personnel performed standardized physical examinations (e.g., grip strength, walking speed, weight, and height) and collected biological samples (e.g., blood, saliva, and nails) during each assessment (Figure 1a,b). In this study, 135 disease‐free participants (with 405 repeated measurements) without prevalent severe chronic diseases (i.e., cardiovascular disease and cancer) across the three waves (waves 2014, 2017, and 2019) were included.

### 2.2 Blood Sample Collection and Storage

A 12 mL whole blood sample was collected from each participant after fasting overnight. Within 1.5 h of collection, samples were centrifuged and aliquoted into six bar‐coded cryogenic vials. One aliquot was used immediately for clinical biochemical analysis and the remaining aliquots were temporarily stored at −30°C for approximately 1 month before being transported on dry ice to the State Key Laboratory of Genetic Engineering at Fudan University, Shanghai, for long‐term storage at −80°C (Liu et al. 2016).

### 2.3 DNA Methylation Measurement and Preprocessing

DNA methylation was measured using the Methylation EPIC v1.0 BeadChips (850 k, Illumina, CA) with 300 μL of blood cell samples, conducted by Sinotech Genomics Co. Ltd. (Shanghai, China). Raw data were processed using the R packages *minfi* and *ChAMP*. To minimize technical bias, samples were randomized across processing chips and plates. Singular value decomposition (SVD) was performed to identify potential batch effects from chip slides and arrays. Samples with > 1% of measured sites showing a detection *p* value > 0.01 were excluded. Probes were filtered based on the following criteria: detection *p* value > 0.05 in ≥ 5% samples; non‐CpG targets; bead count < 3; SNP‐containing probes; and cross‐reactive probes. After quality control (QC) filtering, the remaining probes underwent beta mixture quantile normalization (BMIQ) to correct probe‐type bias. β‐values were calculated as the ratio of methylation signal intensity to total signal intensity for downstream analyses.

To account for variability in blood cell composition, we estimated the proportions of major leukocyte subtypes (i.e., CD8+ T cells, CD4+ T cells, natural killer (NK) cells, B cells, and monocytes) using the online calculator provided by the DNA Methylation Age Calculator (https://dnamage.clockfoundation.org).

### 2.4 Macro‐Phenotypes Measurement

#### 2.4.1 Clinical Biomarkers

Blood biochemistry (e.g., lipid profiles), complete blood count (e.g., platelet count), and other circulating biomarkers (e.g., homocysteine) were measured using standard laboratory techniques at the biochemistry laboratory of Rugao People's Hospital.

#### 2.4.2 Electrocardiography

Following a 5–10 min rest, standard 12‐lead resting electrocardiography (ECGs) were recorded using an MECG‐200 electrocardiograph (MedEx, Beijing, China) with paper speed set at 25 mm/s, standardized calibration (10 mm = 1 mV), and routine filter settings. Measured ECG phenotypes included heart rate, PR interval, QRS duration, V1 S‐wave amplitude (SV1), V5 R‐wave amplitude (RV5), and corrected QT interval (QTc calculated via Bazett's formula).

#### 2.4.3 Body Measurement

Body measurement including weight (kg), height (cm), waist circumference (cm), and hip circumference (cm) was obtained by trained physicians. Body mass index (BMI, kg/m^2^) was calculated as weight divided by height squared. Waist‐to‐hip ratio was derived from waist circumference divided by hip circumference. For the Timed Up and Go test (TUG, sec), participants stood from an armchair, walked 3 m, returned, and sat down, with timed duration measured from backrest release to reseating (Nordin et al. 2008). Walking speed (m/s) was calculated as distance (4‐5 m) divided by time at usual pace. Grip strength (kg) was assessed using a Hand Grip Dynamometer (Shanghai Wanqing Electron Co. Ltd.) with three repetitions per hand, retaining the maximum value across both hands (Ma et al. 2018).

#### 2.4.4 Physical Function

Frailty index was assessed following Fried's phenotype criteria as implemented in prior RLAS studies (Fried et al. 2001; Zhu et al. 2016), including five domains: unintentional weight loss, exhaustion, low physical activity, weakness, and slow gait. Each domain was dichotomized (0 = healthy, 1 = impaired), yielding a composite score from 0 to 5, with lower values indicating better health status.

#### 2.4.5 Mental Function

Cognitive function was assessed using the Revised Hasegawa Dementia Scale (HDS‐R) (Hasegawa 1990), incorporating orientation, calculation, immediate/delayed memory, and general knowledge domains. Total scores range from 0 to 32.5, with higher scores indicating better cognition. Depressive symptoms were assessed using the Chinese version of the 15‐item Geriatric Depression Scale (GDS‐15), with symptom presence defined by reverse scoring of four items (“no” responses) and standard scoring of 11 items (“yes” responses). Total scores range from 0 to15, with higher scores reflecting greater depressive symptom burden (Hou et al. 2020).

### 2.5 Covariates

Chronological age, sex, smoking status (never, current, or previous), drinking status (never, current, or previous), and the estimated proportions of leukocyte subtypes were included.

### 2.6 Statistical Analysis

#### 2.6.1 Participants Characteristics

Categorical variables are described as number (percentage), and continuous variables are described as mean (standard deviation). The chi‐squared (χ^2^) and Kruskal‐Wallis Test were used to assess differences in participant characteristics across the three survey waves for categorical and continuous variables, respectively. A two‐tailed *p* < 0.05 indicated statistical significance.

#### 2.6.2 Identification of Age‐Varying and Age‐Associated CpG Sites

To identify age‐varying and age‐associated CpG sites, we implemented a two‐step analytical approach. In the first step, we fitted two mixed linear models for each CpG site: Model 1 included random intercepts; Model 2 included both random intercepts and random slopes. Both models were adjusted for sex, smoking status, drinking status, and the composition of leukocyte subtypes to mitigate potential biases. We evaluated model fit using the Akaike Information Criterion (AIC). CpG sites where Model 2 significantly outperformed Model 1 were classified as age‐varying. Additionally, if the best‐fitting model (either Model 1 or Model 2) exhibited a statistically significant fixed‐effect *p* value, the CpG site was designated as age‐associated. To account for multiple testing, the Bonferroni correction was applied. In Model 2, the individual‐specific random slope was interpreted as the longitudinal rate of methylation change for each CpG site, reflecting inter‐individual variability in age‐related methylation dynamics.

#### 2.6.3 Regulatory Annotations

Following the identification of distinct CpG site categories, we annotated these sites to genomic features—including First Exon, 5′UTR, Exon Boundary, TSS1500, 3′UTR, gene bodies, Intergenic Region (IGR), and TSS200—and compared the distributional differences across functional genomic regions among these site categories. In addition, we examined the overlap of age‐associated and age‐varying CpG sites with DNase I hypersensitive sites (DHS), which mark open chromatin regions. Enrichment was evaluated using Fisher's exact test based on a contingency table of CpG category × DHS status, and odds ratios (OR) and *p* values were reported.

#### 2.6.4 Associations Between Baseline Risk Factors and Change Rates of CpG Sites

The associations of baseline BMI and socioeconomic status (SES), two well‐established contributors to accelerated aging (Liu et al. 2019), with longitudinal methylation change rates at CpG sites were assessed via linear regression models, adjusted for age, sex, and leukocyte subtype proportions. For each CpG site, statistical significance was evaluated at *p* < 0.05. Fisher's exact test was subsequently used to compare the number of CpG sites showing significant associations with BMI or SES between age‐varying and age‐associated CpG categories.

#### 2.6.5 Calculation of Pace of Aging Across Multiple Organ Systems

Following procedures by Balachandran et al. (Balachandran et al. 2025), we selected macro‐phenotypes associated with the function, or overall health of specific organ systems and with a predefined direction of change with age. These phenotypes were then grouped into eight organ systems. To reduce redundancy, only one representative phenotype was retained when multiple phenotypes reflected the same underlying trait (e.g., BMI was retained over height and weight in the musculoskeletal system). After multiple imputation using the R package *mice* (version 3.18.0), phenotypes that could not be reliably imputed were excluded. The final set included 46 macro‐phenotypes (Figure 1c and Table S1). For each phenotype, a linear mixed‐effects model with random intercepts and random slopes was fitted, adjusted for age, sex, smoking status, drinking status, and the estimated proportions of leukocyte subtypes. The random slope for each participant was used as the phenotype‐specific pace of aging. To derive a composite pace of aging for each organ system, the arithmetic mean of the phenotype‐specific paces of aging was calculated after aligning their directions relative to aging.

#### 2.6.6 Correlation Analysis Between Change Rates of CpG Sites and Organ Systems

The associations between CpG site‐specific paces of aging and the organ system paces of aging were assessed via Pearson partial correlation analysis, adjusted for age, sex, smoking status, drinking status, and leukocyte subtypes proportions, using the R package *ppcor* (version 1.1). Significantly associated CpG sites were selected and annotated to nearby genes.

#### 2.6.7 Pathway Enrichment Analysis

We annotated the significant CpG sites to their corresponding genes using the Illumina MethylationEPIC v1.0 BeadChip manifest file. Functional enrichment analyses were then performed independently across KEGG, GO, and Reactome databases for CpG sites associated with each organ system. To minimize redundancy among enriched terms, pairwise term similarity was calculated within each database. The “Wang” semantic similarity algorithm was applied to GO terms, while the “Jaccard” algorithm was used for KEGG and Reactome terms. Terms with similarity scores greater than 0.5 were retained to construct similarity networks, followed by community analysis using R package *igraph* (version 2.1.4) to partition modules. Within each module, the term with the smallest adjusted *P* value was selected as representative. Finally, non‐redundant terms from GO, KEGG, and Reactome were combined.

## 3 Results

### 3.1 Population Characteristics

Among the 135 participants (aged 70–81 years at baseline), 83 (61.5%) were females and 68 (50.4%) were illiterate. During the three survey waves, no noticeable changes were observed in sociodemographic and lifestyle characteristics such as marital status, smoking, and drinking patterns (Table S2).

### 3.2 Identification of Age‐Varying CpG Sites

**FIGURE 2:**
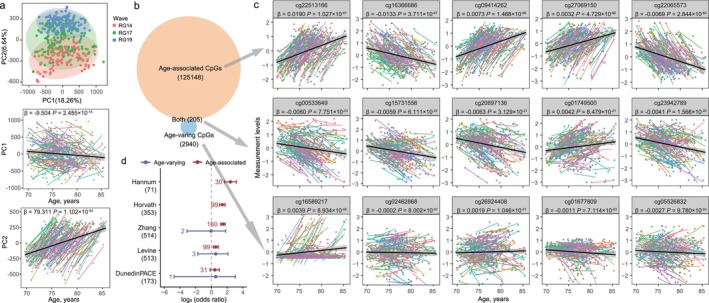
Identification of age‐associated and age‐varying CpG sites. (a) Dot plot showing the PCA distribution of DNA methylation patterns. Different colors represent different survey waves. The longitudinal alterations of PC1 and PC2 with advancing age were analyzed using mixed‐effect models, with lines of different colors representing different participants. (b) Venn diagram illustrating the overlap between age‐associated CpGs (*n* = 125,148) and age‐varying CpGs (*n* = 2940), with 205 CpGs identified as both age‐associated and age‐varying. (c) Longitudinal alterations of five randomly selected CpG sites from each category with advancing age are shown. Mixed‐effect models were used, with lines of different colors representing different participants. (d) Forest plot showing enrichment of CpG sites for five epigenetic clocks in age‐associated and age‐varying CpG sites identified above. The number of CpG sites for each epigenetic clock is annotated in parentheses beneath the name of that clock. The actual number of these clock CpG sites present in our methylation dataset is 64, 327, 508, 510, and 169, respectively. Two‐sided Fisher exact test was used. The *x*‐axis shows the log_2_(odds ratio), with each dot indicating the estimated value and error bars indicating 95% confidence intervals. Different colors represent different CpG categories: Red for age‐associated CpGs and blue for age‐varying CpG sites.

Initial PCA of DNA methylation revealed that both PC1 and PC2 were significantly associated with age (Figure 2a). No significant associations were observed for sex, smoking, or drinking status, although some associations were noted for certain leukocyte subtypes (Table S3). Notably, the association between PC1 and age varied substantially across individuals, whereas PC2 exhibited relatively uniform age‐related trends with minimal interindividual variability (Figure 2a and Figure S1a). This finding suggests that aging is associated with dramatic changes in DNA methylation, with notable inter‐individual variation in certain components such as those captured by PC1.

By fitting mixed‐effect models, we identified 125,353 age‐associated (i.e., sites showing significant shifts in average methylation levels with age) and 3145 age‐varying CpG sites (i.e., sites showing significant inter‐individual variability in methylation trajectories with age) at a Bonferroni‐corrected significance threshold (*p* < 6.0 × 10^−8^; Figure 2b and Table S4). Of these, 205 CpG sites were common to both categories, indicating a small but potentially biologically meaningful overlap between systematic methylation shifts and increasing variability with age. Figure 2c displays the longitudinal alterations of five representative CpG sites from each category with advancing age. Comparing our results with the SATSA longitudinal study (Wang, Pedersen, and Hägg 2018; Wang, Karlsson, et al. 2018), which reported 1316 age‐associated and 570 age‐varying CpG sites, we found that 541 age‐associated CpG sites and 18 age‐varying CpG sites overlapped (Table S5). This modest overlap, particularly for age‐varying CpG sites, likely reflects differences between the studies in population characteristics (e.g., ethnicity and age range), sample size, analytical strategies, and the duration and frequency of follow‐up.

Furthermore, we examined the contribution of each CpG category to PC1 and PC2 by comparing the absolute loadings of age‐varying and age‐associated CpG sites across the two PCs. Age‐varying CpG sites showed significantly larger loadings on PC1 than on PC2, whereas age‐associated CpG sites exhibited higher loadings on PC2 than on PC1 (Figure S1b).

### 3.3 Existing Epigenetic Clocks Included Limited Number of Age‐Varying CpGs

To evaluate whether established epigenetic clocks preferentially incorporate age‐associated or age‐varying CpG sites, we performed an enrichment analysis of age‐varying and age‐associated CpGs for five widely used epigenetic clocks (i.e., HannumAge (Hannum et al. 2013), GrimAge (Lu et al. 2019), Zhang clock (Zhang et al. 2019), PhenoAge (Levine et al. 2018), and DunedinPACE (Belsky et al. 2022)). After correcting for multiple testing, three clocks—HannumAge, GrimAge, and the Zhang clock—showed significant enrichment for age‐associated CpGs. However, none of the existing clocks exhibited significant enrichment for age‐varying CpGs (Figure 2d). This indicates that, although some clocks may include age‐varying CpGs, they, by design, favor CpG sites with strong average associations with age, and thus are not enriched for age‐varying CpGs.

### 3.4 Pathway Enrichment Analysis Reveals Distinct Biological Roles for Age‐Associated and Age‐Varying CpG Sites

**FIGURE 3:**
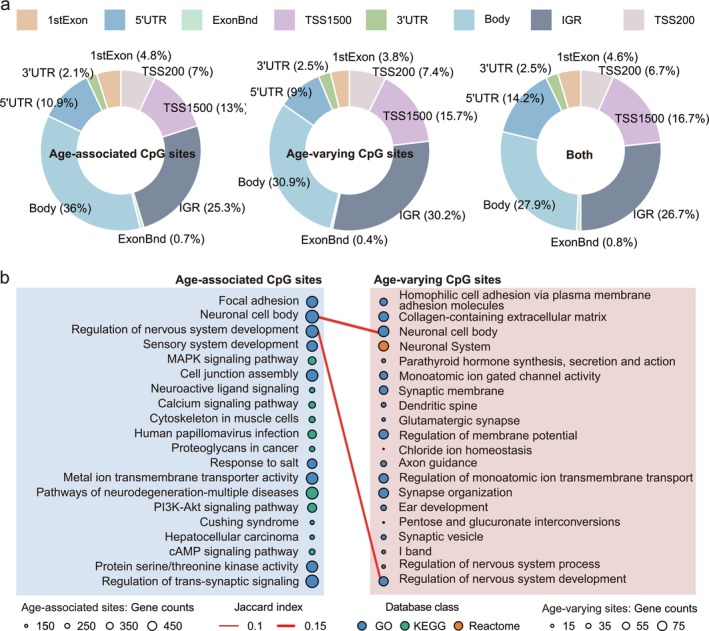
Genomic distribution and pathway enrichment of age‐associated and age‐varying CpG sites. (a) Ring diagram showing the distribution of age‐associated and age‐varying CpG sites across different regions of genomic location. The percentages indicate the proportion of each category within respective genomic location. (b) Pathway enrichment analysis results for age‐associated and age‐varying CpG sites. The size of the circles indicates the number of genes involved in each pathway, while the color indicating the dataset from which the pathways were derived. Red lines connect common pathways enriched by both age‐associated and age‐varying CpG sites, with line thickness representing the Jaccard index between these pathways.

To characterize these CpG sites from a functional genomics point of view, we first determined their distribution within the different regions of genomic location. There was no significant relationship between both age‐varying and age‐associated CpGs and their respective genomic location (Figure 3a). We further examined whether age‐associated and age‐varying CpG sites differ in their enrichment in DHS. Age‐varying CpGs were significantly more enriched in DHS regions than age‐associated CpGs (OR = 1.17, *p* = 3.85 × 10^−5^; Figure S2).

Pathway enrichment analysis revealed that both age‐associated and age‐varying CpG sites are involved in diverse biological processes, albeit with distinct functional profiles. Age‐associated sites were enriched in pathways related to nervous system development (e.g., regulation of nervous system development, sensory system development), cell communication (e.g., MAPK and calcium signaling pathways), cytoskeletal organization in muscle cells, and pathways linked to age‐related diseases (e.g., human papillomavirus infection and proteoglycans in cancer). Age‐varying sites were predominantly enriched in pathways related to cell adhesion, neuronal development and function, synaptic complexity, ion channel activity, and organ development (Figure 3b and Tables S6 and S7). Notably, both categories showed significant enrichment in nervous system‐related pathways including regulation of nervous system development and neuronal cell body, suggesting that the nervous system is a central target of both systematic and variable epigenetic changes during aging (Oh et al. 2025).

Interestingly, despite the lack of broad pathway enrichment (Table S8), several of the 205 overlapping CpG sites are located in genes known to play critical roles in key aging‐related processes and organ system functions. For example, *ZFHX4* (cg02298862) and *RBM38* (cg18523477) are involved in DNA damage response, signal transduction, and DNA binding processes central to aging and age‐related diseases such as cancer (Zhang et al. 2014). Additionally, *PCDHGA* (cg08616061) and related genes are implicated in the formation and function of cell–cell junctions in the brain, suggesting potential roles in neural aging and cognitive decline. These findings suggest that the overlapping CpG sites may possess unique biological significance, potentially reflecting sites where consistent population‐level changes coincide with high interindividual variability—features that could mark regulatory loci with complex roles in aging.

We further evaluated associations of BMI and SES with longitudinal methylation change rates at age‐associated and age‐varying CpG sites. Both factors were associated with methylation change rates at a larger number of age‐varying CpG sites compared with age‐associated CpG sites (Figure S3), indicating differential sensitivity of these two CpG categories to well‐established risk factors.

### 3.5 CpG Sites Reflecting the Pace of Aging Across Multiple Organ Systems

**FIGURE 4:**
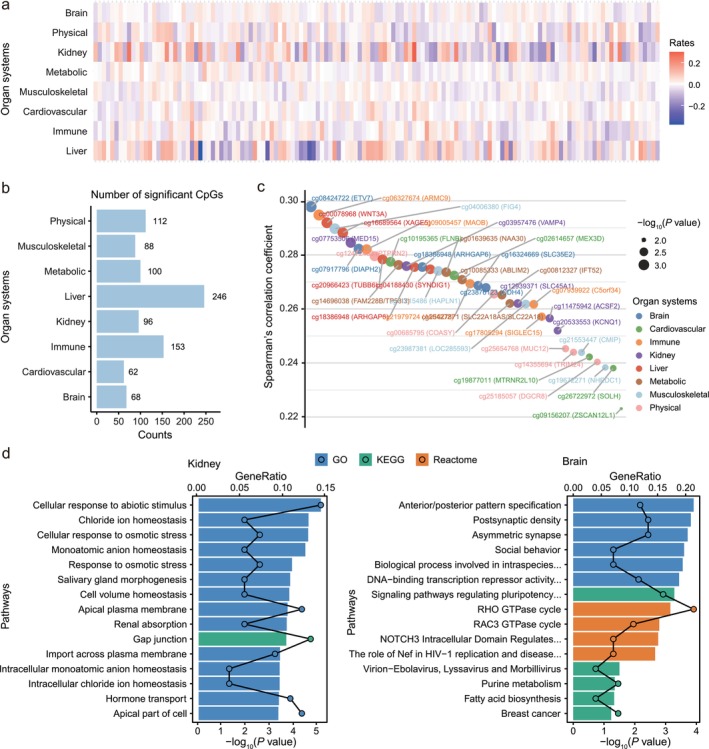
Analysis of change rates of age‐varying CpG sites and the pace of aging across eight organ systems. (a) Heatmap showing the heterogeneous pace of aging across different participants and organ systems. Each row represents an organ system, and each column represents a participant. The color gradient indicates the direction and magnitude of aging rates: Red signifies positive associations (accelerated aging), while blue signifies negative associations (decelerated aging). (b) Bar chart displaying the number of CpG sites significantly associated with the pace of aging for each organ system. (c) Scatter plot showing the top five CpG sites for each organ system. The color of the points represents different organ systems, and the size of the points indicates the significance of the association between the CpG site and the organ system's pace of aging. (d) Pathway enrichment analysis results for CpG sites whose longitudinal change rates were associated with the pace of aging in the kidney (left) and brain (right). Pathways are color‐coded by source database: Blue for Gene Ontology (GO), green for KEGG, and orange for Reactome. Circles positioned along the top axis according to its gene ratio. The *x*‐axis shows the −log_10_(*p* value), and the *y*‐axis lists various biological pathways.

The pace of aging exhibits heterogeneity across individuals and organ systems (Figure 4a), reflecting both personalized and system‐specific aging trajectories. We next examined whether interindividual variation in longitudinal methylation change rates at CpG sites captures differences in organ‐specific aging, focusing on age‐varying CpG sites only according to our previous two‐step analytical approach. We identified 925 significant associations between the longitudinal rates of change at age‐varying CpG sites and the pace of aging across multiple organ systems (*p* < 0.05, Figure 4b and Table S9). The liver exhibited the highest number of linked CpG sites, whereas the cardiovascular system had the fewest (Figure 4b). Figure 4c displays the top five genes associated with each organ system. Some of these genes have been shown to be involved in hallmarks of aging, as detailed in Table S10.

Pathway enrichment analysis revealed that CpG sites associated with the kidney and brain were enriched in biological pathways relevant to each system's function, whereas CpG sites assigned to other organ systems did not exhibit similarly clear within‐system enrichment patterns (Tables S11–S18). Specifically, CpG sites associated with kidney aging were enriched in pathways such as chloride ion homeostasis, cellular response to osmotic stress, and monoatomic anion homeostasis—processes critical for renal fluid and electrolyte regulation. CpG sites associated with brain aging were enriched in pathways such as postsynaptic density and asymmetric synapse—structures essential for synaptic transmission and neuronal plasticity (Figure 4d). These system‐specific patterns highlight potential intervention targets to preserve system‐specific functions during aging. Intriguingly, some CpGs associated with one organ system were enriched in pathways typically linked to other systems—for example, musculoskeletal system‐related CpGs enriched in pathways associated with cardiovascular function (e.g., circadian rhythm) and neuronal aging (e.g., serotonergic synapse). This cross‐system overlap highlights the interconnected nature of aging and potential interorgan influences (Bartsch et al. 2015; Kirk et al. 2025; Kumar et al. 2024; Tian et al. 2023).

## 4 Discussion

Leveraging longitudinal DNA methylation data from a Chinese cohort, we identified two distinct types of CpG sites, which were termed as age‐associated and age‐varying CpG sites, based on their dynamic patterns during aging. Although both types of CpG sites are involved in biological pathways closely associated with aging and the functioning of organ systems (e.g., the neuronal cell body pathway for the nervous system), they also exhibit distinct functional implications. By mapping the associations of age‐varying CpG sites with the pace of aging across eight organ systems (e.g., brain and kidney), we further identified organ/system‐specific associated CpG sites whose corresponding genes were enriched in biological pathways relevant to their respective organ/system functions (e.g., the response to osmotic stress pathway for kidney function).

Many CpG sites included in established epigenetic clocks based on cross‐sectional data (Field et al. 2018; Crimmins et al. 2021; Hannum et al. 2013; Horvath and Raj 2018; Levine et al. 2018; Li et al. 2013; Horvath 2013) exhibit limitations in capturing true dynamic changes associated with aging. This limitation may partially explain the substantial heterogeneity observed across existing epigenetic clocks in terms of CpG composition, age associations, and predictive performance for health outcomes (Horvath and Raj 2018; Liu et al. 2020). These discrepancies suggest the existence of at least two types of CpGs, age‐associated and age‐varying CpGs (Field et al. 2018). Studies based on cross‐sectional data are therefore likely capturing age‐associated CpGs or a mixture of both types, rather than isolating their unique contributions. In contrast, longitudinal data offer the potential to disentangle them, as they may reflect different aspects of the aging process and capture distinct health risks. Despite previous studies that have attempted to use longitudinal changes in phenotypes for model training (Belsky et al. 2022), they still fail to capture site‐specific methylation dynamics at the individual level. In contrast, this study leverages true longitudinal DNA methylation data to systematically identify and distinguish between age‐associated and age‐varying CpG sites and to explore their potentially divergent biological functions. We further demonstrated that both methylation types are associated with specific health outcomes and the functioning across multiple organ systems, providing clues for understanding the diverse molecular mechanisms underlying aging and laying a foundation for future development of personalized assessments and interventions targeting organ and system aging.

When comparing our findings with the SATSA longitudinal study (Wang, Pedersen, and Hägg 2018; Wang, Karlsson, et al. 2018), we observed a modest overlap, particularly for age‐varying CpG sites. This pattern indicates that age‐associated changes may be relatively conserved across populations, whereas age‐varying methylation is more sensitive to cohort‐specific factors and analytical strategies. Future larger and more harmonized longitudinal methylation studies will be important for delineating the reproducible core of age‐varying CpG changes and for clarifying the sources of inter‐cohort heterogeneity.

Our analysis suggests that age‐associated and age‐varying CpG sites may drive aging dynamics through distinct yet complementary mechanisms, collectively contributing to the multidimensional representation of biological aging. Specifically, while both types are enriched in well‐established aging‐associated pathways (e.g., cell adhesion (Colucci et al. 2025)), their predominant functional annotations differ substantially. Age‐associated sites are primarily associated with pathways reflecting intrinsic biological aging processes, while age‐varying sites show a more pronounced enrichment in pathways related to the structural and functional integrity of physiological systems. For instance, several pathways associated with the functioning of the nervous system have been identified, including neurotransmitter transport, axon guidance, and cognition‐related pathways. These pathways play critical roles in maintaining neural circuit integrity and supporting higher‐order brain functions. Notably, despite these functional divergences, a degree of convergence between the two types was also observed at specific functional nodes. For instance, they all enrich in pathways linked to the neuronal cell body, suggesting a potential point of interaction or shared regulation in brain aging and therefore highlighting the nervous system potentially serve as a central target of both systematic and variable epigenetic changes during aging.

The genes harboring organ system‐specific associated CpG sites were functionally relevant to their corresponding organs/systems, indicating that biological methylation sites may exert directional and organ‐restricted regulatory effects on the aging progression. Nevertheless, within‐system pathway enrichment emerged predominantly for the brain and kidney. Both organs have been reported to exhibit relatively strong tissue‐autonomous aging programs: the brain undergoes tightly regulated epigenomic remodeling linked to neuronal maintenance and synaptic function (Jeong et al. 2025; Oh et al. 2025), whereas the kidney shows age‐dependent alterations in metabolic and detoxification pathways that map closely onto declining renal physiology (Fang et al. 2020). In contrast, systems such as the immune and metabolic systems are shaped predominantly by systemic, cross‐organ regulatory processes—including chronic inflammation, endocrine signaling, and circulating metabolic cues, which may dilute organ‐specific signals and result in weaker within‐system enrichment. Further studies are needed to elucidate the biological basis of these differences.

Several limitations should be acknowledged. First, the participants in the current analysis were limited to older adults. Although this age group represents a critical stage of life in which cumulative biological damage and functional decline become most apparent, it remains unclear whether the identified methylation signatures are consistently manifested across broader life‐cycle stages, and we cannot capture the population‐level trajectory of methylation changes across the entire lifespan. Future studies incorporating longitudinal epigenetic data from more diverse age groups will be essential to characterize a more comprehensive dynamic nature of aging‐related methylation patterns. Second, the relatively limited sample size may have constrained statistical power to detect more significant CpG sites and precluded robust inference on potential causal relationships. Third, we cannot completely rule out the influence of potential sources of noise (e.g., technical variability) on the observed age‐varying CpG sites. Nevertheless, the high reproducibility of Illumina methylation array, standardized sample collection and storage protocols across all three waves, and concurrent processing of all longitudinal samples under identical laboratory conditions enhance confidence in the reliability of our findings. Fourth, due to interpopulation genetic and environmental heterogeneity, the findings derived from this Chinese cohort may not be directly transferable to other ethnic or racial groups. Moreover, the limited availability of independent longitudinal DNA methylation datasets precluded external validation of the identified methylation signatures, thereby constraining the broader applicability of our results. Finally, DNA methylation is a highly tissue‐specific molecular feature. The current analyses were based on blood‐derived DNA methylation profiles, yet different tissues may reflect distinct regulatory mechanisms underlying systemic aging.

## 5 Conclusion

In conclusion, leveraging longitudinal DNA methylation data from a Chinese cohort despite its limited numbers of older adults, this study reveals the differential yet complementary regulatory mechanisms underlying the different DNA methylation dynamics during aging. Distinguishing between age‐associated and age‐varying methylation sites provides deeper insights into the heterogeneity of biological aging across individuals, offering a novel foundation and potential targets for personalized interventions to organ and system aging and age‐related diseases. The potential utility of age‐varying CpG sites in constructing next‐generation epigenetic clocks is an exciting avenue for future research. Larger cohorts and improved mechanistic understanding will be essential to accurately interpret the biological directionality of these CpG sites and to harness their potential in clock development.

## Author Contributions

Z.L. designed and supervised the study. X.J. and H.C. analyzed the data. X.J., H.C., and Z.L. interpreted the results. X.J., H.C., and L.Z. drafted the manuscript. All authors revised the manuscript. Z.L. took responsibility for the content of the article. All authors have read and approved the submitted version of the manuscript.

## Funding

This work was supported by grants from the National Natural Science Foundation of China (82171584 and 82401856), Research Center of Prevention and Treatment of Senescence Syndrome, School of Medicine Zhejiang University (2022010002), Hangzhou Meilian Medical Co. Ltd. (Kheng‐20241116, to ZL), “Pioneer” and “Leading Goose” R&D Programs of Zhejiang Province (2023C03163 and 2025C02104), Zhejiang Key Laboratory of Intelligent Preventive Medicine (2020E10004), and Zhejiang University School of Public Health Interdisciplinary Research Innovation Team Development Project. The funders had no role in the study design; data collection, analysis, or interpretation; in the writing of the report; or in the decision to submit the article for publication.

## Ethics Statement

The Rugao Longitudinal Aging Study (RLAS) was approved by the Human Ethics Committee of the School of Life Sciences of Fudan University, Shanghai, China (No: BE1815).

## Consent

Informed consent was provided by every participant or his/her relatives.

## Conflicts of Interest

The authors declare no conflicts of interest.

## Supporting information


**Figure S1:** Distributions of individual age slopes and CpG loading differences between PC1 and PC2.
**Figure S2:** Enrichment of CpG categories in DNase I hypersensitive sites (DHS).
**Figure S3:** Associations of baseline BMI and SES with longitudinal change rates of CpG sites.

**Table S1:** Phenotypes used to calculate the pace of aging across multiple organ systems in the Rugao Longitudinal Ageing Study.
**Table S2:** Characteristics of the 135 participants across three survey waves in the Rugao Longitudinal Ageing Study.
**Table S3:** Associations of PC1 and PC2 with age and covariates in linear mixed‐effects models (with both random intercept and random slope).
**Table S4:** Identification of age‐associated and age‐varying CpG sites in Rugao Longitudinal Ageing Study.
**Table S5:** Overlap of age‐associated and age‐varying CpG sites identified in the present study with those reported in the SATSA cohort (Wang, Karlsson, et al. 2018; Wang, Pedersen, and Hägg 2018, Epigenomics).
**Table S6:** Enriched biological pathways for age‐varying CpG sites.
**Table S7:** Enriched biological pathways for age‐associated CpG sites.
**Table S8:** Enriched biological pathways for the 205 CpG sites common to both age‐associated and age‐varying categories.
**Table S9:** Correlations between change rates of CpG sites and pace of aging across multiple organ systems in Rugao Longitudinal Ageing Study.
**Table S10:** Top Organ‐associated CpG‐linked genes implicated in aging mechanisms.
**Table S11:** Enriched biological pathways for age‐varying CpG sites whose change rates were associated with the pace of aging of kidney.
**Table S12:** Enriched biological pathways for age‐varying CpG sites whose change rates were associated with the pace of aging of brain.
**Table S13:** Enriched biological pathways for age‐varying CpG sites whose change rates were associated with the pace of aging of cardiovascular system.
**Table S14:** Enriched biological pathways for age‐varying CpG sites whose change rates were associated with the pace of aging of immune system.
**Table S15:** Enriched biological pathways for age‐varying CpG sites whose change rates were associated with the pace of aging of liver.
**Table S16:** Enriched biological pathways for age‐varying CpG sites whose change rates were associated with the pace of aging of metabolic system.
**Table S17:** Enriched biological pathways for age‐varying CpG sites whose change rates were associated with the pace of aging of musculoskeletal system.
**Table S18:** Enriched biological pathways for age‐varying CpG sites whose change rates were associated with the pace of aging of physical system.

## Data Availability

Only deidentified participant data will be available upon approval of a proposal and signing of a data access agreement. Researchers with a specified purpose should send a request email to Prof. Zuyun Liu. The codes for statistical analyses of this study will be available upon request from Prof. Zuyun Liu.
