# Supplementary material for: Longitudinal Profiling of DNA Methylation Reveals Age‐Varying CpG Sites and Novel Insights Into Aging Heterogeneity
Source: Aging Cell. 2026 Jan 2;25(1):e70362. doi: 10.1111/acel.70362 (PMC12759111; doi:10.1111/acel.70362)
Supplement: Supplementary file 1 — Figure S1: Distributions of individual age slopes and CpG loading differences between PC1 and PC2. Figure S2: Enrichment of CpG categories in DNase I hypersensitive sites (DHS). Figure S3: Associations of baseline BMI and SES with longitudinal change rates of CpG sites. [file ACEL-25-e70362-s002.docx]

**
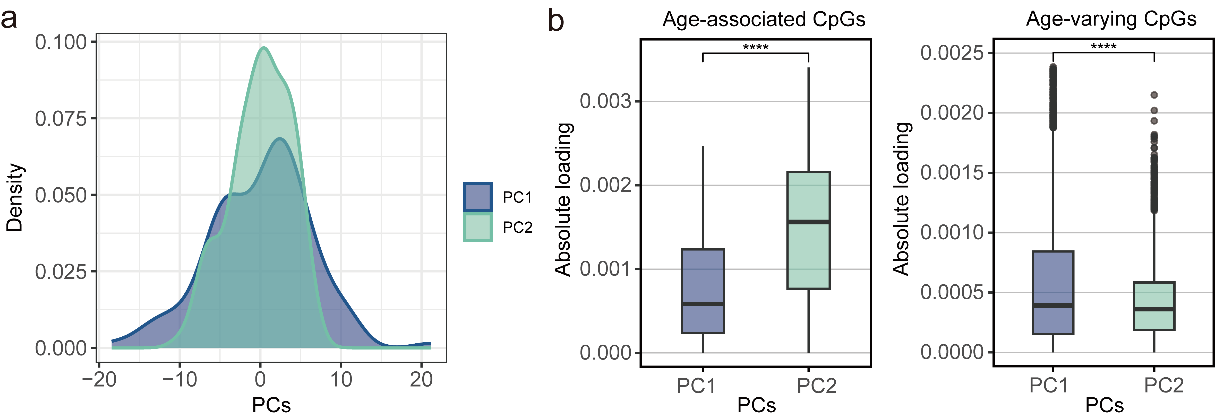
**

**Figure S1. Distributions of individual age slopes and CpG loading differences between PC1 and PC2.**

**a**. Density distributions of individual-level age slopes for PC1 and PC2. Slopes were estimated using linear mixed-effects models adjusting for sex, smoking status, drinking status, and the composition of leukocyte subtypes. **b**. Boxplots comparing the absolute loadings of age-associated (left) and age-varying (right) CpG sites on PC1 and PC2. **** denotes p <= 0.0001 (Wilcoxon test).


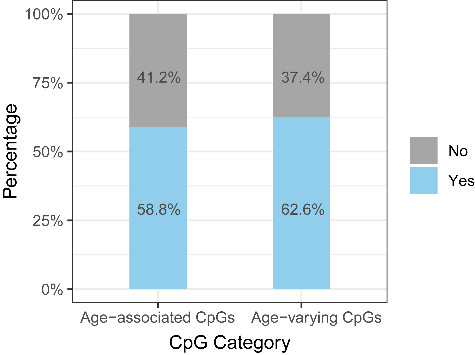


**Figure S2. Enrichment of CpG categories in DNase I hypersensitive sites (DHS).**

Bar plot showing the proportions of age-associated and age-varying CpG sites overlapping DNase I hypersensitive sites (DHS).


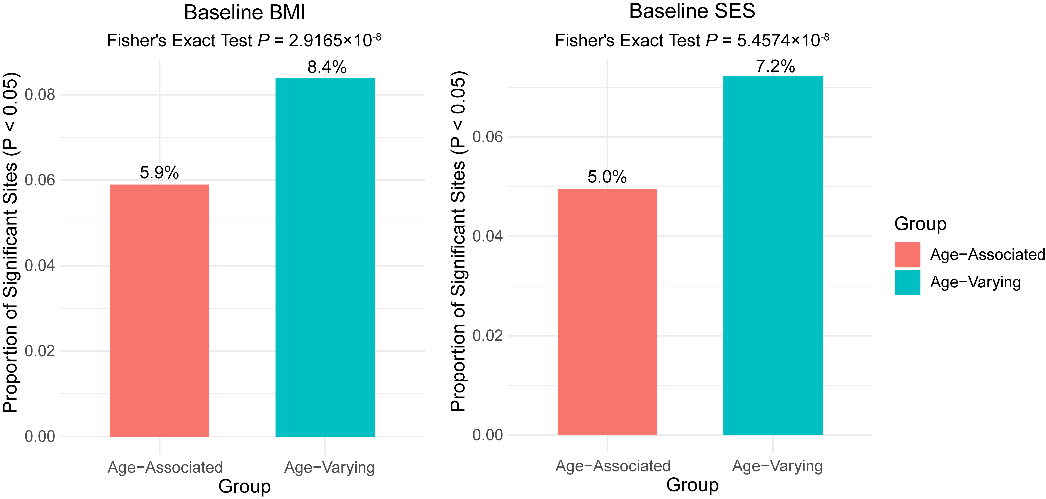


**Figure S3. Associations of baseline BMI and SES with** **longitudinal change rates of CpG sites.**

The bar plots display the proportions of CpG sites showing significant associations (*P* < 0.05) between baseline body mass index (BMI; left panel) or socioeconomic status (SES; right panel) and longitudinal methylation change rates, stratified by CpG category (age-associated vs. age-varying). Associations were estimated using linear regression models adjusted for age, sex, and estimated leukocyte subtype proportions. Fisher’s exact test was used to compare the proportion of significant CpG sites between the two categories, with *P* values shown above each panel. SES was derived from a composite score combining educational attainment and wealth ownership indicators. BMI, body mass index; SES, socioeconomic status.
